# Annotations

**Published:** 1887-07-02

**Authors:** 


### Annotations.

Careless Dispensing.

Frances Macdonald has just died at the Colney Hatch Asylum in consequence of a dose of dilute carbolic acid given to her by mistake. Walter Dendy, who has been dispenser at Colney Hatch sixteen years, was preparing a carbolic lotion, when a prescription for a draught for Frances Macdonald was handed to him. Dendy immediately began to prepare the draught, and after putting in, as may be supposed, the drug prescribed, filled the bottle up with what he doubtless imagined was water. It was carbolic acid and water in about equal parts. Nothing need be said to aggravate the pain which Dendy probably feels. But it is more than unpleasant to think that the like may happen any day outside an asylum. It ought not to have been possible for such an accident to take place. It cannot be too much to expect that one set of vessels shall be used for those preparations which we are intended to take into our stomachs, and another set for those we are intended to apply externally. Certainly Dendy was culpable in using for a mixture a vessel in which he had previously placed carbolic acid. Familiarity tends to breed contempt among dispensers; but just as signalmen and others in positions of great responsibility should make it one of their chief duties to fight against that contempt which comes of familiarity, so should chemists: and they should so order their work that accidents of such a kind should be as impossible as the administration of a poisonous crystal like that of chloral hydrate instead of an innocent powder like that of rhubarb. The dispensing of drugs is as responsible a duty as that of commanding an army in the field.

The Growth of the Sunday Fund.

The amounts collected at the various churches and chapels continue to be announced, and there are hopeful indications that a substantial increase will be shown in the total. If that should prove to be the case the reason may be twofold. Some people have probably increased their contributions by way of celebrating the Queen's Jubilee. The larger number have doubtless done it because the claims of the hospitals have been urged upon them with greater force and clearness than heretofore by the meetings held during the Hospitals Fortnight. It is difficult to imagine how even a few persons manage to deceive themselves into the idea that the Hospital Sunday services and collections may have done as much harm as good. It is really a question of whether advertising does or does not promote the growth of a business. If it does not, then Holloway, Day and Martin, and a hundred others have been very stupid, and the fortunes they are said to have amassed have existed only in the imagination. If the anti-Sunday Fund people are prepared to maintain this contention nothing more need be said. They probably know that the famous Bishop Berkeley, as the result of much speculation and reading of philosophical systems, came to the conclusion that there was no such thing as "matter." Upon which it was wittily observed, "If the bishop says there is no matter, it does not matter what the bishop says." If the anti-Sunday people are prepared to maintain the thesis that advertisement and publicity are not of service in the raising of money for good objects, they must be so hopelessly ignorant of the manners of the times that it really does not matter anything about their opinions. They are probably prepared to maintain that the angles at the base of an isosceles triangle are unequal, or any other proposition equally absurd.

Doctors "out in the cold."

To some persons State honours are a very serious business. Many a poor rich man would rather be Sir Gorgius Midas than Savonarola or St. Paul. It is wonderful how names and titles impose upon people who read little and think less. There is no doubt that many doctors are seriously unhappy because none of their number have ever been raised to the peerage in this country. Bishops are peers for life, and sit in the House of Lords. The Archbishop of Canterbury is the first subject in the realm. In the same way lawyers have elbowed their way to the front, and still pass from the Woolsack to a hereditary peerage. Poor doctors have never been able to get beyond a baronetcy. A little knowledge of history, with the slightest dash of a philosophical temperament, enables us to contemplate the apparent want of State courtesy to medical men with considerable equanimity. When the Church was poor she did not dwell in kings' houses. When she became powerful kings and statesmen had to make what terms they could with her, and those terms often included the raising of her clergy to the highest posts of authority in the State. Similarly the lawyers were a necessary part of Government. No State could exist without a legal system, and a legal system implied a separate class learned in the art and mystery of the law, a class whom the kings often required to manipulate for their own purposes, but who could only be manipulated on certain terms and conditions. The practice of the law tends to make a man bold and overbearing, as may be seen in the solicitor's clerk, who can browbeat a defenceless widow with the greatest courage and resolution. Ages of legal practice have enabled lawyers to raise themselves to a level almost with the highest in the State. There is no very great objection to this. Medicine, however, as a really learned profession is comparatively modern. The doctor is no necessary part of the government of the State, as the churchman has been, and as the lawyer still is and must continue to be. He has no means of making terms with the monarch or his minister; and so, unless civilisation should assume a distinctly higher type, he will probably continue to be esteemed as of less moment than the pushing lawyer, the rich brewer, or the successful blacking-maker.

